# Urban living labs as innovation infrastructure for local urban intervention acceleration and student social learning: the impacts on community wellbeing in Heerlen

**DOI:** 10.3389/fpubh.2023.1242151

**Published:** 2024-01-05

**Authors:** Stefano Blezer, Nurhan Abujidi, Herwin Sap

**Affiliations:** Smart Urban Redesign Research Center, Zuyd University of Applied Sciences, Heerlen, Netherlands

**Keywords:** urban living labs, social learning, placemaking, urban intervention, co-creation, community wellbeing, urban innovation

## Abstract

Cities are championing urban experiments in order to address societal challenges and increased urban complexity. In fact, and following fellow researchers, urban experiments are used as a method in a broader trend in public policy to align urban planning with citizen needs by viewing cities as platforms for societal transformation that require, and should draw on, active involvement of residents. In this study, we demonstrate the impacts of placemaking and Urban Living Labs not only for healthy environments but also in facilitating transdisciplinary learning. Therefore, we elaborate on the Aurora transformation process in the neighborhood GMS in Heerlen-North as being one of the 16 Dutch neighborhoods that need extra attention to its socio-urban challenges due to the historical context and consequent local development. Hence, providing two main results for ULLs as infrastructure for innovation for community wellbeing. First, as an alternative spatial planning approach for urban contexts with extreme social-urban conditions that draw on the multitude of local values to generate and accelerate urban transformations going beyond the traditional impacts of urban transformations including public health equity, health outcomes, and addressing social-economic determinants of community wellbeing. Second, as an infrastructure for education innovation encompassing and operationalising social learning theory. Subsequently, it addresses societal issues in these neighborhoods, such as loneliness, social exclusion, or democratic decision-making more appropriately and enhances student, and urban stakeholder, learning through transdisciplinary collaboration among those involved and by connecting education, research methods and questions, and real-life socio-urban challenges. We conclude the article by emphasizing its novelty, providing a discussion, and enumerating implications for theory, practice, policy, and research.

## 1 Introduction

Contemporary cities are going through an intense transformation phase driven by increasing urban complexity and grand societal challenges. Hence, there is a growing trend in public policy to align urban developments to citizens' needs by viewing cities as platforms for societal transformation toward addressing issues such as inclusion, equity, and human development opportunities [see, e.g., (1, 2)].

To overcome the issues related to complex societal challenges and rigid, sectoral planning, cities are engaging with innovative solutions and championing urban experiments in order to deliver on these challenges [see, e.g., (3, 4)]. Diverse approaches to work with such urban complexity go beyond the conservative, incremental practices of planning that are normally regulative and normative. To fill this gap in theory and practice, more experimental bottom-up and engaging approaches surfaced such as co-creation or participatory planning, i.e., placemaking, for which Urban Living Labs (ULLs) are used as a method for the active involvement of residents and other relevant stakeholders in healthy human development [see, e.g., (5–7)].

In this study, we will focus on the articulation of the innovation and added value of both placemaking and the ULL set-up to demonstrate the impacts of such approaches not only for vital, healthy, and inclusive environments but also in facilitating a transdisciplinary learning environment for students, researchers, and other urban stakeholders with a focus on social learning theory.

## 2 Context

The neighborhood GMS in Heerlen-Noord is acknowledged by the National Government as one of the 16 priority neighborhoods in the Netherlands for its severe and urgent urban challenges, such as energy poverty, low literacy, and cultural diversity (8). These urban challenges are chronical and have been deeply rooted in their local historical context, i.e., the coal mines closure in the 1960–1970s and consequent socio-urban challenges such as unemployment, low income rates, aging population, and drug-related nuisance leading to a strong negative image (stigma) on the area. In fact, these socio-urban concerns have significant implications for public health equity and community wellbeing. For example, the difference between the life expectancy of higher and lower socio-economic status inhabitants in the Netherlands is 6 years, and the difference in *healthy* years of life between them is even 15 years (9, 10), not to mention this difference increased due to COVID-19 (11).

Within this context, there are diverse concentrations and clusters of multiple urban challenges. One of them is the Aurora apartment building, a social housing unit of over 200 dwellings housing a community from over 60 nationalities. The housing block has been renovated recently and painted with the largest artistic mural in Europe. This development aligns with national targets for housing associations to upgrade their housing stock energy efficiency wise as well as the present development perspective of the municipality to enhance the quality of the urban environment by emphasizing culture and arts, while in parallel enhancing its local identity in link to its distinctive local qualities and historical context.

To come short, it is important to, amidst the challenges faced by neighborhoods like GMS, understand and examine the interplay between, on the one hand, urban development approaches and strategies and, on the other hand, public health equity and community wellbeing to improve both the quality of the built environment and the social-health fabric of these neighborhoods. Consequently, we asked ourselves the following research question:

“How can Urban Living Labs as an (urban) innovation and learning infrastructure contribute to vital, healthy and inclusive neighborhoods by physical and socio-spatial interventions in neighborhoods like GMS in Heerlen-North?”

**Figure 1:**
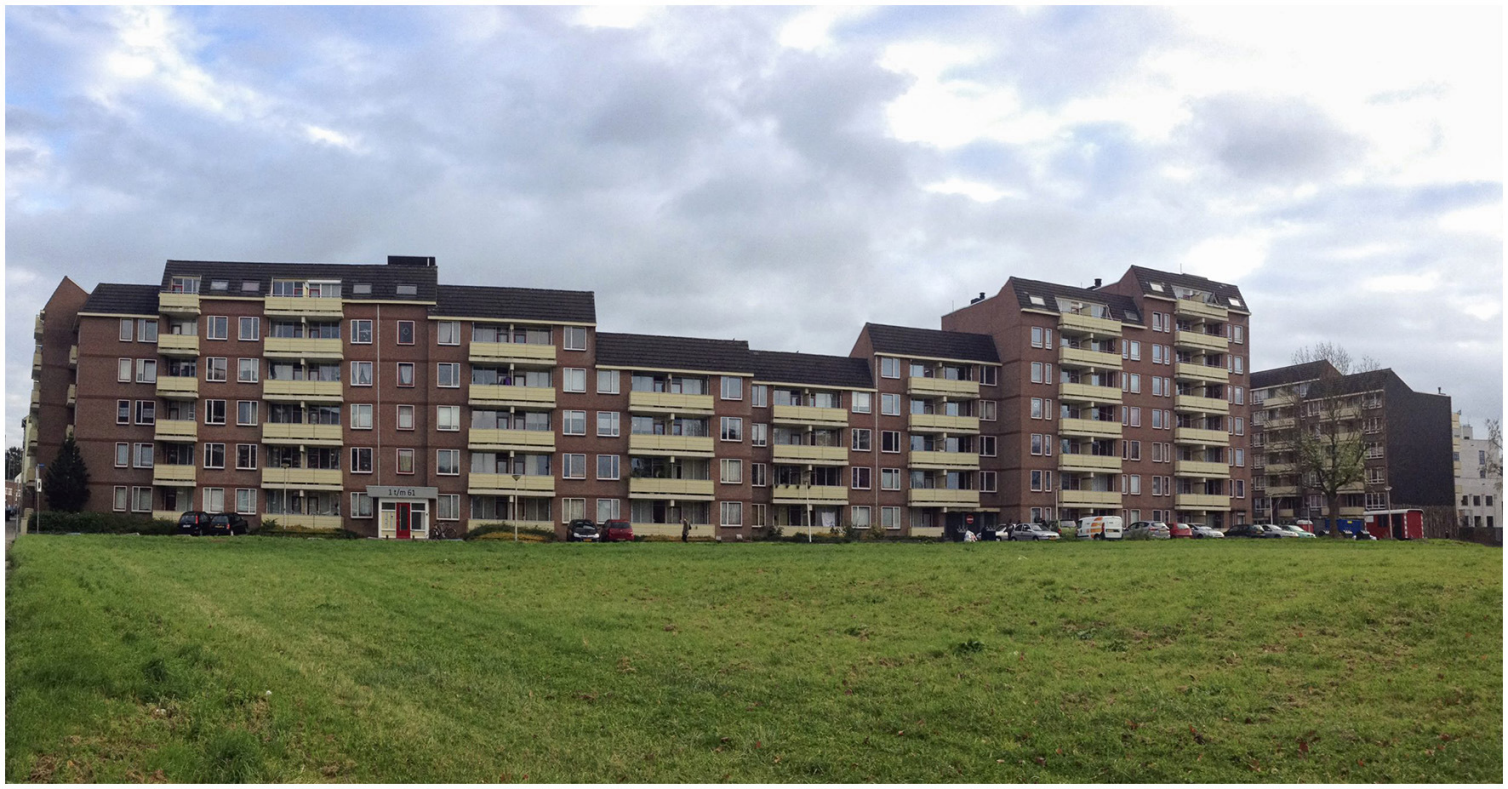
Aurora apartment building before painting and renovation (Source: Boa Mistura).

**Figure 2:**
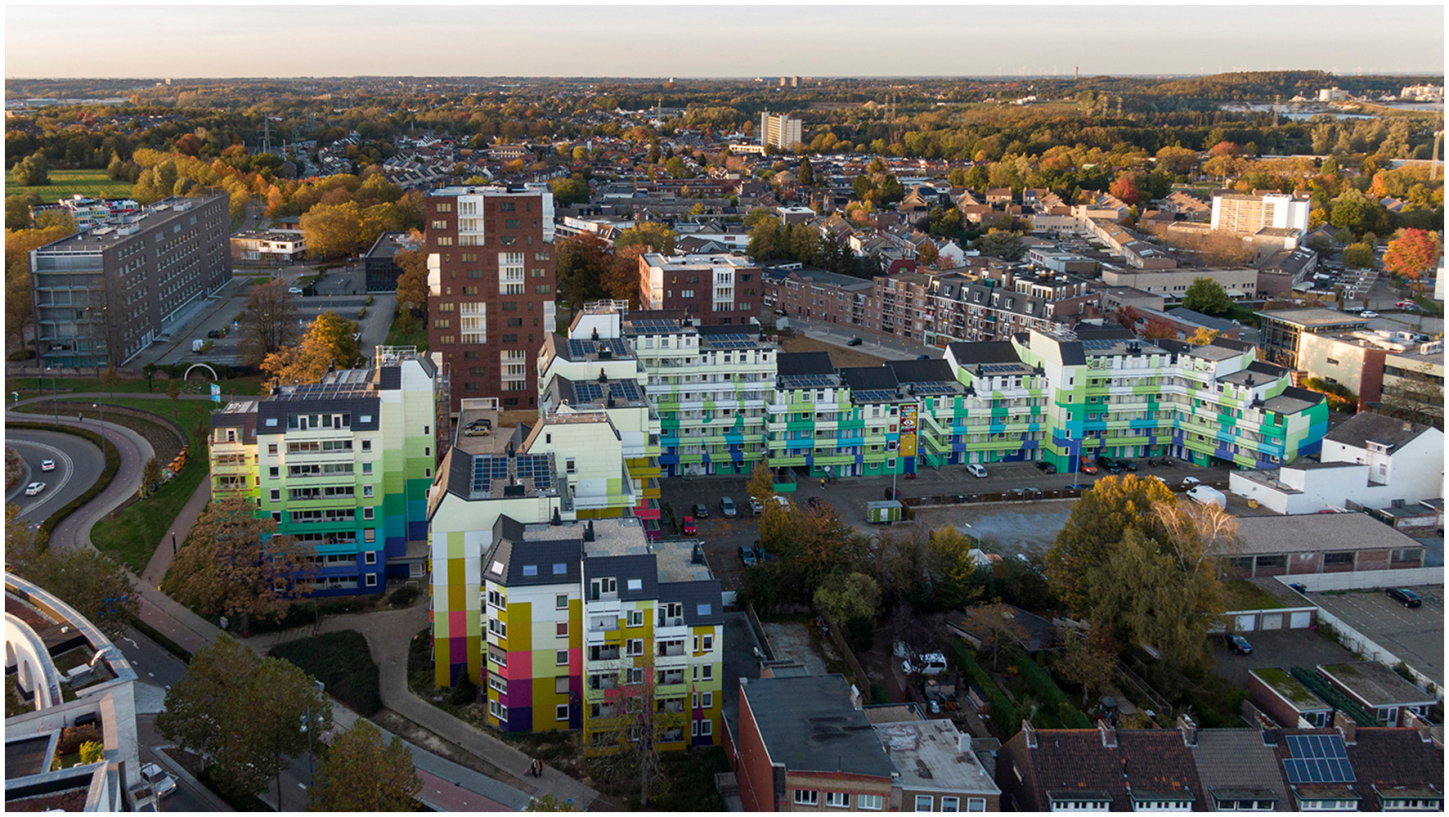
Aurora apartment building after painting and renovation (Source: Boa Mistura).

**Figure 3:**
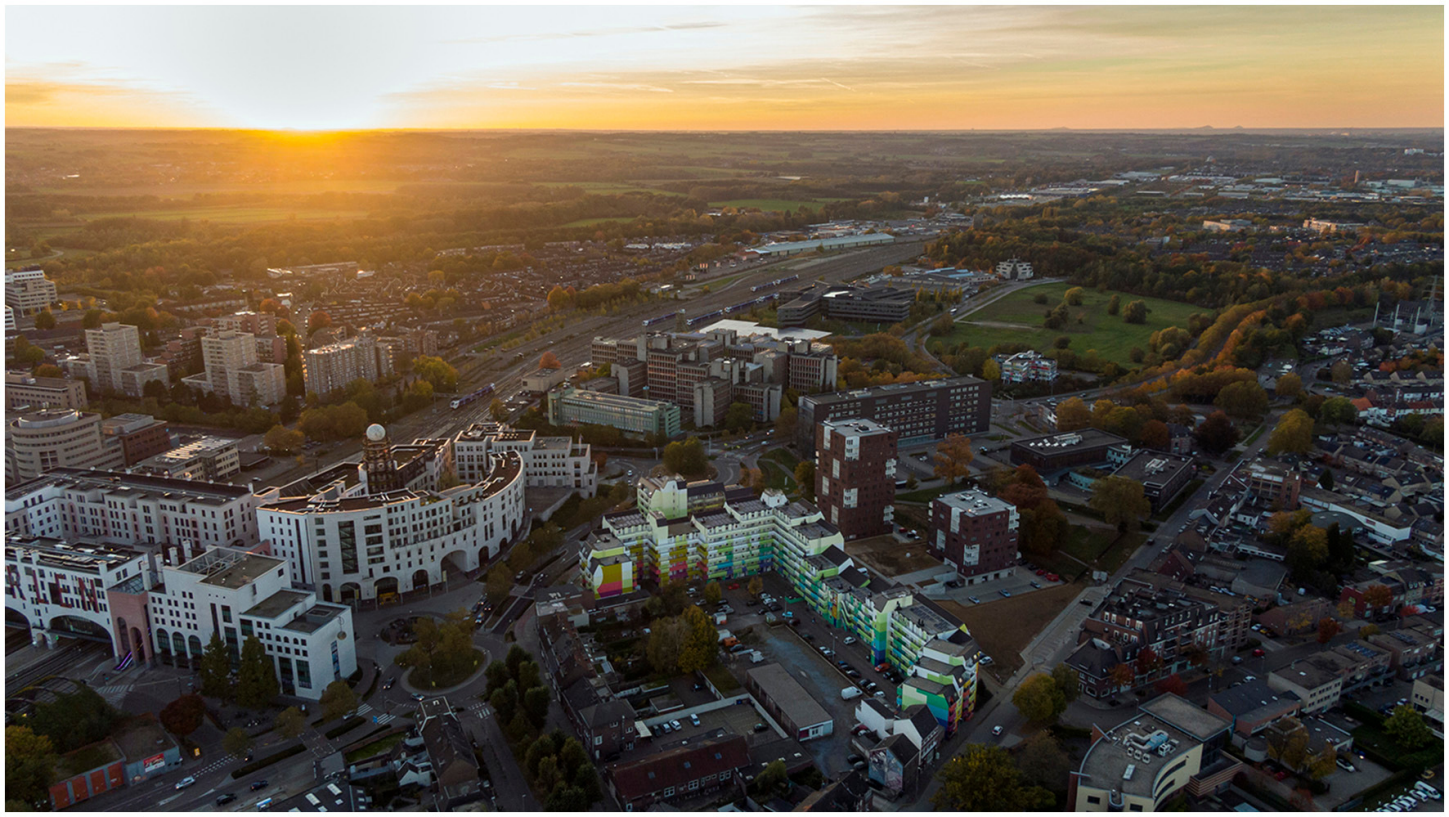
Aurora apartment building and GMS neighborhood incl. the Maankwartier station area (Source: Boa Mistura).

For illustration purposes, Figures 1–3 show the location and its context.

## 3 Placemaking and urban living labs

According to Marrades et al. (12), placemaking in urban planning emerged as a response to the inefficiency of other planning approaches and as a reconceptualisation of how city sites are constituted and urban transformation takes place. As such, it offers an approach to bridge the gap between exchange value (economic profits) and user value (daily life activities). Placemaking also gives the chance to respond to urgent short-term needs of the local community and provide a direction to the long-term structural transformation. From a spatial planning perspective, placemaking articulates urban sites as “places” prioritizing and responding to demands from communities and focusing first and foremost on people (12). Additionally, it may be defined as an incremental way to improve the quality of a place over a long period of time with context-specific experimental interventions and activities (13). Consequently, it is seen as a process of creating “quality places” that people want to live, work, play, and learn in (13) and that creates an attachment or connection between the community and the place they live in, also referred to as their sense of place (14).

Fincher et al. (15) stress the importance of local lived experiences and everyday encounters in placemaking for professional urban planners in order to overcome the gap between exchange and user value. ULLs provide the potential to overcome this gap as they are not only concerned with the place under study but exist in relation to its historical, institutional, spatial, and temporal dimensions while seeking transformation (12). In this way, ULLs can be understood as city sites that provide a learning arena within which the co-creation of innovation can be pursued between local stakeholders and community actors (16). Rather than achieving a pre-determined objective *per se*, the focus is on learning (17) as a means through which experiments, i.e., urban interventions, become successful because urban experimentation is “*fluid, open-ended, contingent and political”* [(18), p. 260] and centers people in the urban planning process and fosters the relation between those people and their places. As such, ULLs are transdisciplinary in nature and advance “place understanding” through a process of collaboration and interactive learning among urban stakeholders. This distinguishes them from neoliberal methods of planning as they are capable of meaningfully remake public space into places that are co-designed and reimagined by a community and local stakeholders while existing in relation to its context using placemaking as a concept and philosophy to urban and spatial planning. In the Dutch context, this may seem especially relevant in neighborhoods like GMS in Heerlen due to their “vulnerability” and cultural and migration diversity because of its temporary, zoning and exclusion policy approach that creates a monoculture causing loss of potential to urban vitality [see, e.g., (19, 20)].

## 4 Placemaking, urban living labs, and the healthy city

Recently, Horstman and Knibbe (9) have shown that the healthy city concept is about public space, social exclusion, urban vitality, and social interactions in the city. They do state that:

“*The living environment of people with low incomes often contains more health threats than the public space of high income groups'* [p. (21); own translation].

These authors, for example, refer to criminality, (noise) nuisance, dilapidation, less greenery, air pollution, or fewer social encounter opportunities to support their claim. Hence, three perspectives on the built environment and the healthy city arise:

1. Public space for social interactions in the built environment. Here, the concept of “public familiarity” (21) is important as it emphasizes that social interactions increase feelings of safety and familiarity consequently leading to less social isolation in cities (22).
2. Public space for connectedness to one's neighborhood. This perspective links with the theories of placemaking, sense of place and thoughts of Jane Jacobs about city diversity, urban vitality, and its implications on human wellbeing, such as feelings of safety, ownership and community. Indeed, placemaking is a successful social movement (9) though one should note its criticisms that places may be viewed as an economic product too much [see, e.g., (23, 24)].
3. Social mixing in the built environment. Here, it is proposed that mixing certain socio-economic inhabitant groups automatically leads to more physical quality of the living environment, more social cohesion and increased social capital. However, research has shown the opposite of homogenous neighborhoods (25) and that social mixing assumptions pay too little attention to the historical context of places and the aspirations of community members (26).

Placemaking, as a conceptual shift in urban planning, not only reshapes the physical environment but also reimagines the social fabric of these neighborhoods. Additionally, ULLs advance place understanding and placemaking through a process of collaboration and interactive learning among local stakeholders. Also, research in the public health domain has repeatedly shown the importance of, for example, social interactions and community engagement to address health disparities rooted in the (re-)production of the built environment. Hence, examining how ULLs can function as innovation infrastructure by providing a social learning arena is essential in the pursuit of community wellbeing impacts in neighborhoods.

## 5 Urban living labs and social learning

Sustainability challenges are visible on a global level, while sustainability transitions happen on a local level due to the fact that innovations and interactions between stakeholders are situated and understood by those involved on a local scale (27). In the urban context, sustainability transitions are about changes in markets, policy, culture, technologies, and infrastructure as well as in human behaviors and practices [see, e.g., (28)]. In the urban context, sustainability challenges are about achieving the SDGs that require widespread diffusion of technological innovations and new infrastructures [see, e.g., (29)]. Against this background on the need for innovation actions on a local scale, while having sustainability challenges on a higher scale, it is important (if not a necessity) to develop the needed and appropriate knowledge, practices, and expertise to achieve the required innovation actions to govern those changes and achievements.

ULLs arose for their potentials for collective learning and exchange of ideas about the built environment and its ecosystem (30). Yet, their understanding on how to facilitate local sustainability transitions remains limited (31). Meanwhile, the potentials of Universities of Applied Sciences have been recognized internationally and in ULL innovation literature to prepare students and stakeholders through the use of ULLs to address issues in society transdisciplinary and in line with the SDGs in context [see, e.g., (32, 33)]. In fact, sustainability transitions require new forms of education and pedagogical tools that enable students and professionals to deal with rapid changes, increasing complexity, criticizing knowledge and uncertainty (34), also in urban innovation transformations. To that end, higher education institutes play a crucial role because they are locally rooted and globally connected (35) being able to educate location-aware global citizens (36, 37). The social learning theory therefore gained renewed interest in education literature as learning takes place via accumulated knowledge, and (inter)personal and vocational professionalization (38) in an environment where collaboration, critical thinking, and co-creation are centered (37).

The social learning theory encompasses four elements arguing that engagement with social issues is fundamental to how learning takes place and how people become who they are (38). Drawing on these four elements enhances student learning impacts as it intertwines personal and professional development with locally relevant societal issues. It also provides possibilities for transversal (students from different years in one discipline) and multi-level (vocational, bachelor, and master) collaboration and learning in addition to multidisciplinary perspectives. The elements are as follows:

1. *Learning as belonging:* Students are part of the ULL community; they learn within and with the local stakeholders and community actors. Therewith, engagement in and understanding of actual societal challenges can lead to the development of relevant knowledge, competencies, and skills.
2. *Learning as becoming:* Students collaborate with the ULL community to develop their own work identity relative to other disciplines. Therefore, professionals develop a broader understanding and knowledge base of the complexity and interconnectedness of actual urban questions.
3. *Learning as experience:* Students learn by working on real-life societal issues in a local context. The context, and its undergoing changes, makes learning and working meaningful for students. This will help to bridge the gap between abstract concepts and context-specific questions and needs. Hence, the city environment becomes the campus.
4. *Learning as doing:* Students learn to take initiative and responsibility regarding societal issues and the ULL community to gain knowledge and skills and reflect on their own as well as other disciplines. Being active in the development, implementation, and monitoring of certain interventions on the local scale bridges the gap between diverse scales that students work on. From neighborhood to building or product scale, resulting in more interrelated hands-on knowledge development.

## 6 Results: Aurora apartment building design co-creation process

The Aurora apartment building area has gone through a process of transformation since the coal mine closure that left a strong stigma on the building and the inhabitants, being a residence and area for mainly underprivileged communities and a place for drugs and prostitution. Many efforts and projects have been implemented in the past 10 years to transform the place and bring better life quality and imago by the housing association Wonen Limburg. Within this context of ongoing transformation, it was an important step to work on the development of the courtyard of the Aurora apartment building to enhance the sense of community and the creation of a social place that is safe, climate-adaptive, and healthy for the tenants.

The Aurora apartment building courtyard is a parking lot for the tenants of the social housing association. However, the parking lot is only rented out for two-thirds of its capacity and the residents experience the courtyard as a site for illegal activities (ranging from illegal parking to drug usage), and as a non-inviting stressing environment (i.e. not climate resilient and physically closed). Hereto, Wonen Limburg and the research center Smart Urban Redesign (SURD) developed a co-creation process to identify residential needs and wishes for the Aurora apartment building courtyard to transform it into a climate-resilient, circular, and community place that supports the residents in their daily life activities; a place where sense of community and collective identity can flourish. Consequently, to improve community wellbeing, urban vitality and social cohesion as impacts.

**Figure 4:**
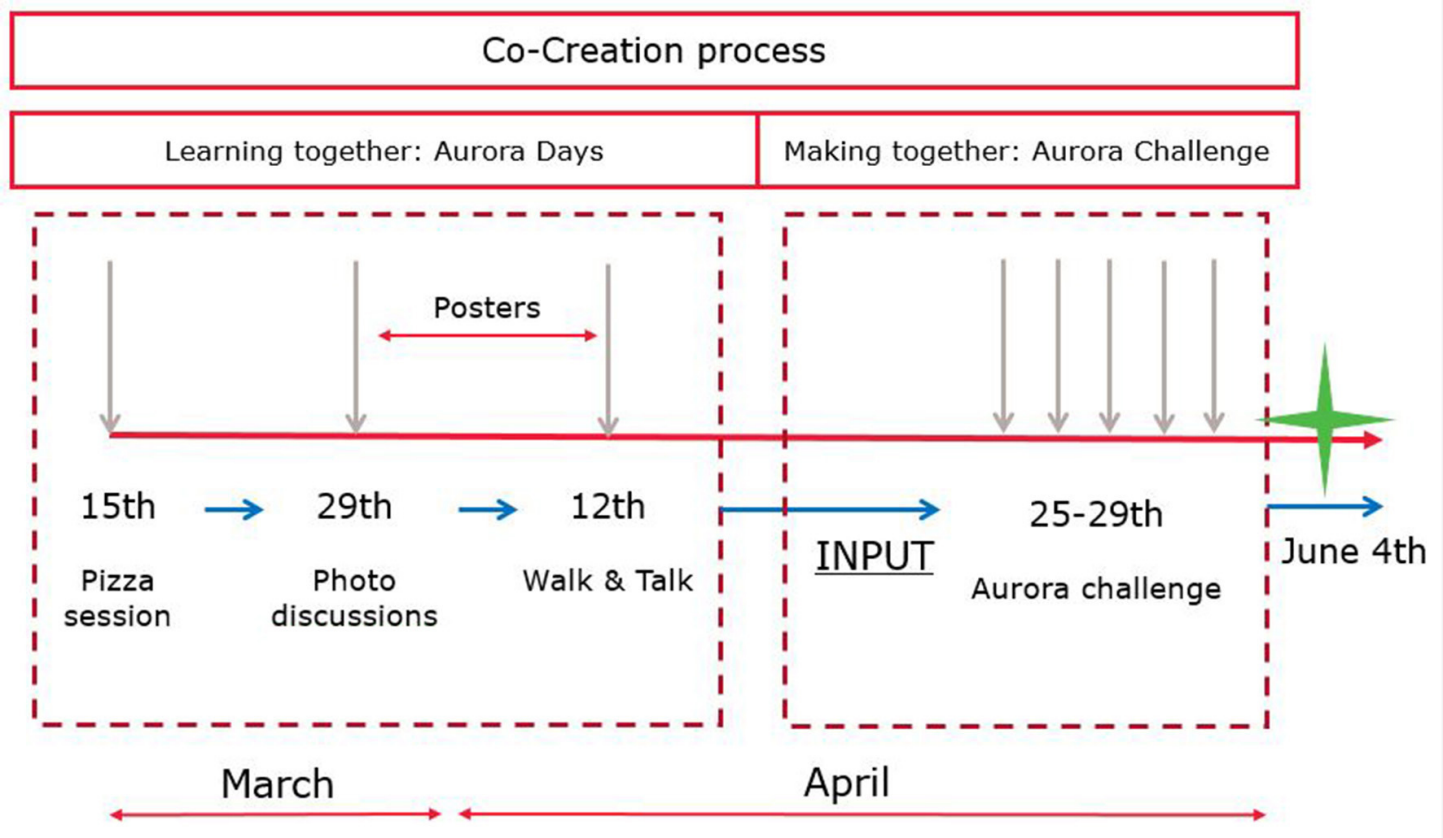
Aurora apartment building intervention co-creation process (Source: SURD).

In this process, both learning together and making together as part of co-creation (17) are outlined in the Aurora Days and Aurora Challenge, respectively. Both worked toward the Spektakeldag festival on 4 June 2022 in which the housing association opened the largest mural of Europe festively (postponed earlier due to COVID19 measurements). In Figure 4, the co-creation process is illustrated.

The Aurora Days included an informal pizza session, photo group discussions, and Walk and Talk sessions. These were held on March 15th, March 29th, and April 12th in 2022. These served (1) to get to know the inhabitants and for them to get familiar with the SURD students and staff, (2) to gain insights into eight different locations around the area that concerns, both positive and negative, the community and that were emphasized by the community members in conversations during the pizza session, and (3) to enhance place understanding of the direct context around Aurora with urban experiences from the community members, respectively. In the photo group discussions and Walk and Talk sessions, activities revolved around target groups, i.e., children, older adults, mothers, singles, and migrants. Therefore, in-between result posters were hung up at all four the entrances of Aurora apartment building to allow for adjustments, additions, and refinements by residents themselves. The Aurora Days were coordinated by a Built Environment and an Occupational Therapy intern duo at SURD and were supported and facilitated by researchers, teachers, fellow students, citizens, and Wonen Limburg.

The Aurora Challenge was a multidisciplinary, transversal, and multi-level design challenge held in the Aurora apartment building. The Aurora Challenge took place in the week of 25 April 2022 to 29 April 2022. Four international and interdisciplinary student groups worked for 1 week non-stop on the design challenge to translate the collected insights from the Aurora Days into an urban design intervention. The students ranged from first-year BSc students to second-year MSc students and came from the Netherlands, Iran, and Germany and the spatial planning, civil engineering, building technology, transportation, occupational therapy, and nursing disciplines. The Aurora Challenge led to a winning design, voted for by the residents, Wonen Limburg, and local stakeholders that was showcased at the Spektakeldag festival on June 4th in 2022.

## 7 Results: Aurora apartment building active experimentation and intervention implementation

Since the Aurora Challenge, the involved stakeholders have been working toward the implementation of the winning design scenario. The implementation was done in two phases. First, an active experimentation of the winning design elements on, and after, the Spektakeldag festival. Second, and based on the monitoring impacts after the Spektakeldag festival, the actual intervention implementation in the Aurora courtyard.

**Figure 5:**
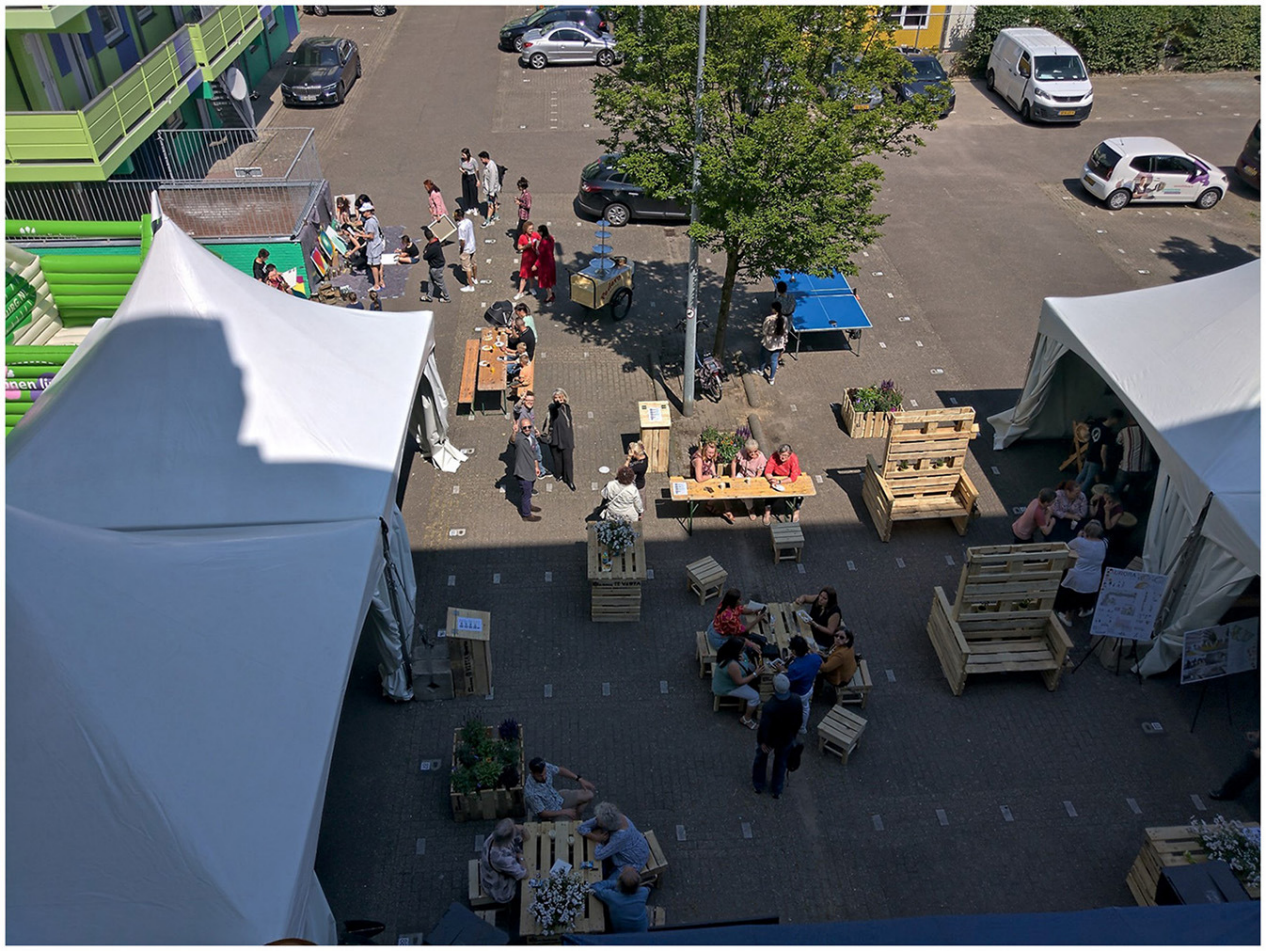
The Spektakeldag active experimentation June 2022 (Source: SURD).

The active experimentation phase included the making and testing of circular street furniture, trash bins and planters from the winning design that were made after the Aurora Challenge in collaboration with the local trash and recycling company (RD4) and the local vocational education institution (VISTA college). This circular furniture introduced the inhabitants of Aurora to a particular sustainability challenge, i.e. circular economy, in an appropriate way. The method for introducing citizens to sustainability challenges is especially important in “vulnerable and culturally diverse” neighborhoods as shown earlier by Abujidi et al. (19) in the city of Kerkrade-West, the Netherlands. The circular furniture was showcased and tested at the Spektakeldag festival (see Figure 5).

Since then, the impacts and use of the circular products have been monitored by a MSc. Architecture intern at SURD who lives in the Aurora apartment building. The intern has mapped the frequency, duration, and type of activity that took place around the circular furniture as well as if citizens used the circular furniture in “unexpected ways.” To illustrate, it was observed that some children were climbing on the circular furniture that was otherwise assumed not being outside at all. Consequently, it resulted in new forms of appropriation, physical movement and sports, and social activities and interactions between children and their parents.

**Figure 6:**
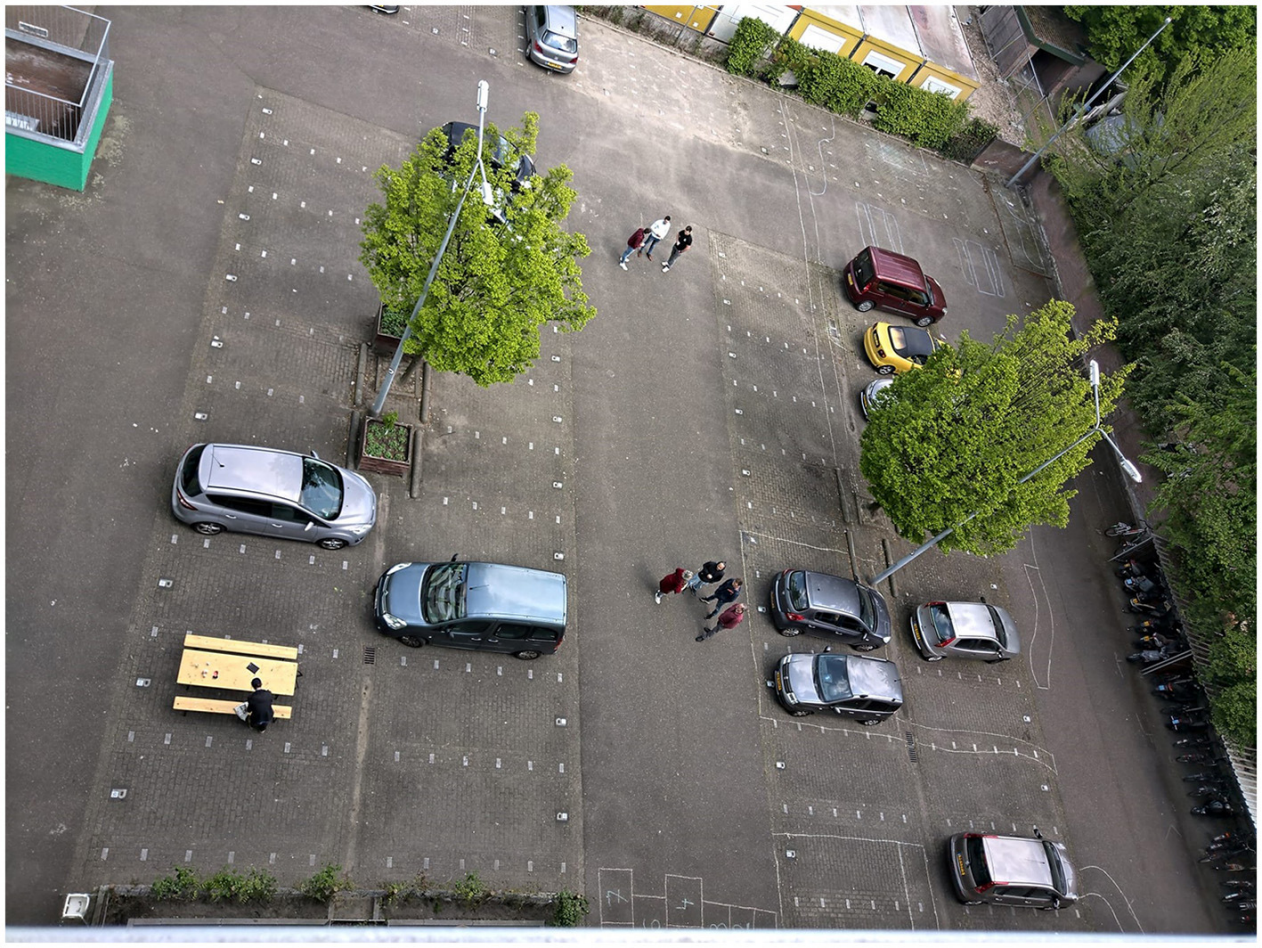
The Aurora apartment building courtyard in March 2022 (Source: SURD).

**Figure 7:**
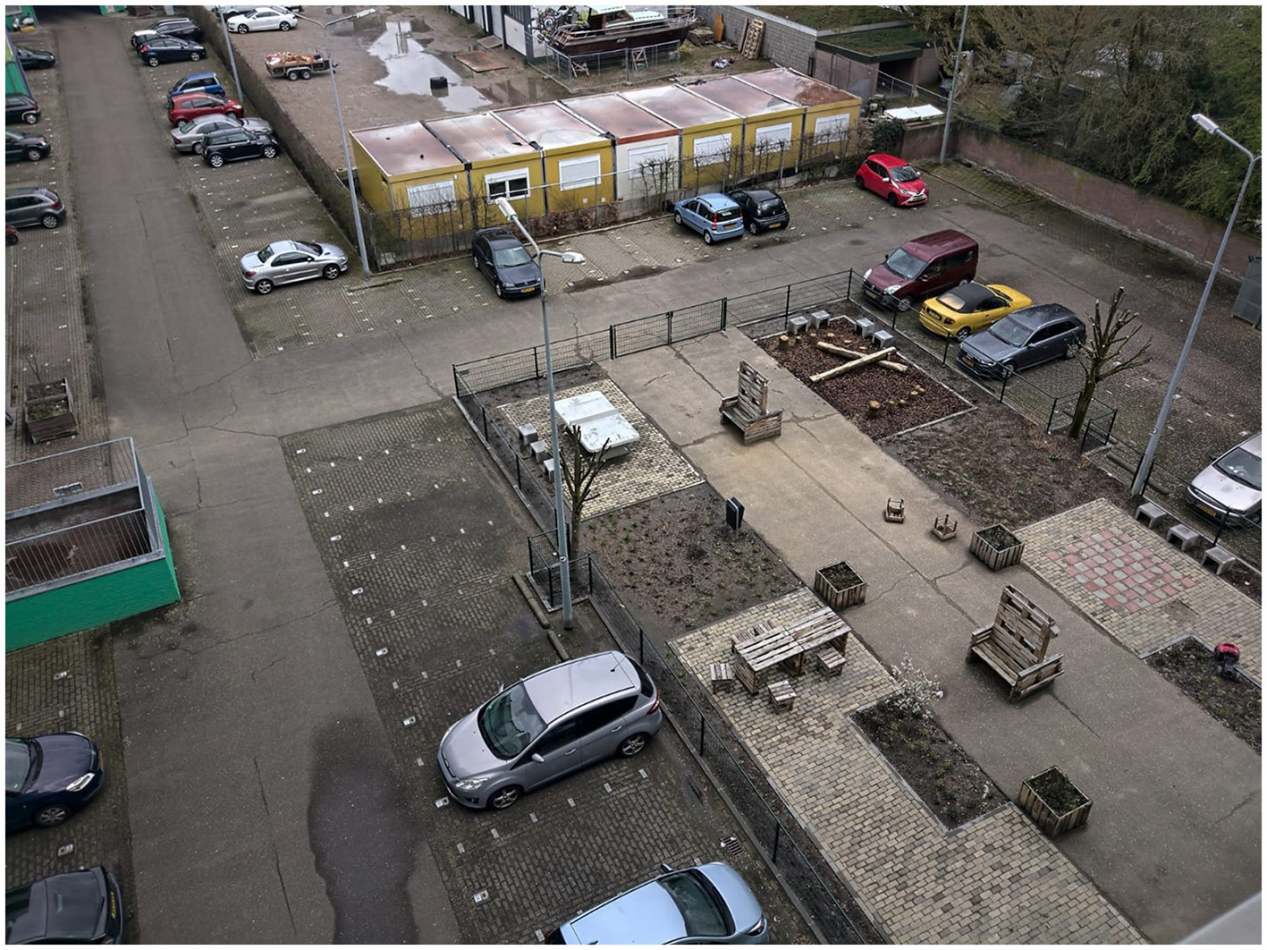
The intervention implementation result April 2023 (Source: SURD).

**Figure 8:**
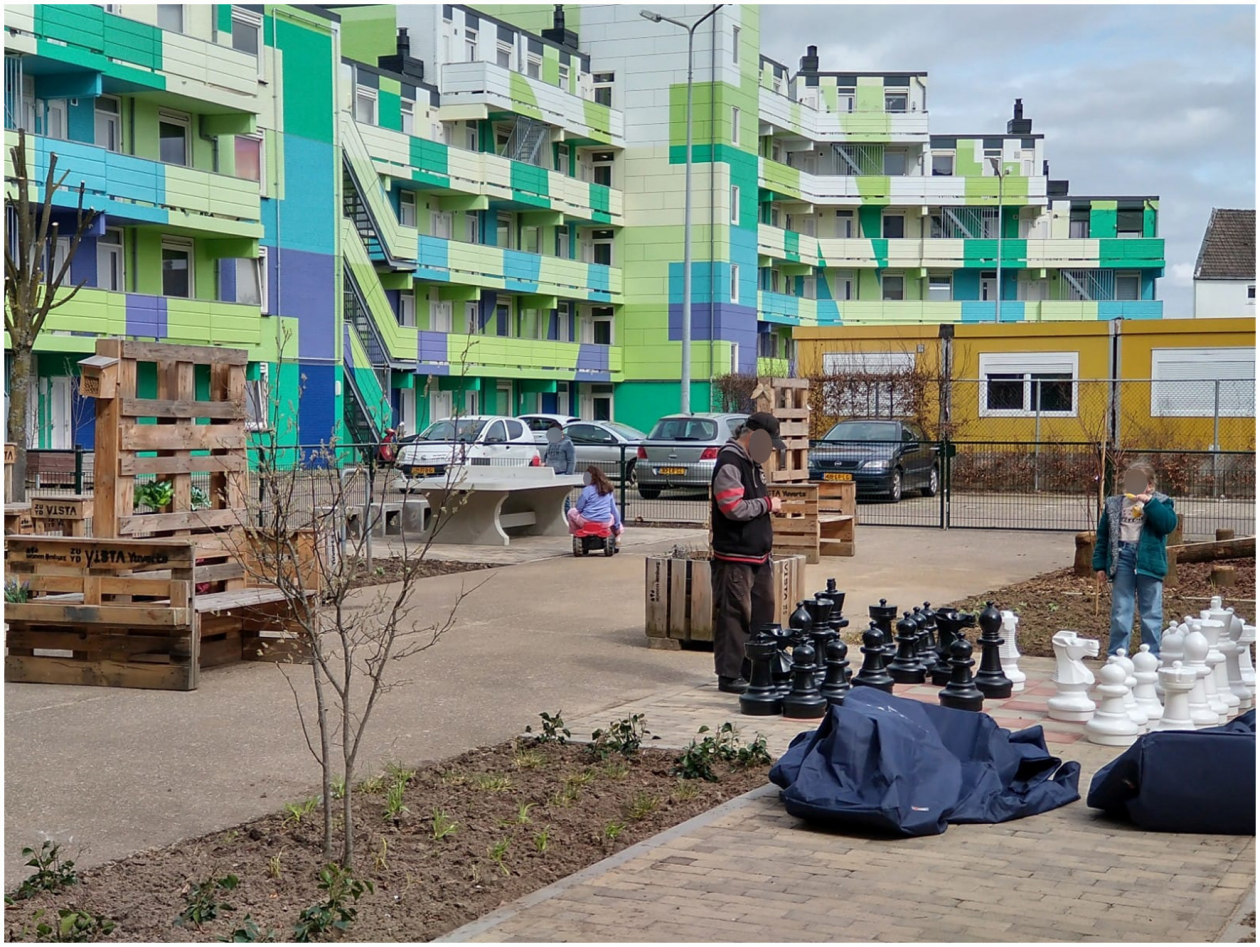
The intervention implementation impacts April 2023 (Source: SURD).

The intervention implementation phase started with the social housing association and a local landscaping company with jobs for people at a distance from the labor market. They captured the essence of the winning design and the experiences and insights from the impact monitoring process to develop the new Aurora apartment building courtyard. As of April 2023, the Aurora courtyard completed its physical transformation of the parking lot. The new Aurora courtyard now includes sitting places, circular furniture, edible greenery (e.g., mint leaves), a chess board, a ping pong playing field, and nature-based relaxing areas (see Figures 6–8 for the old and new situation; Figures 7, 8 taken in spring period 2023).

## 8 Results: social learning for students and stakeholders in practice

As explained earlier, the social learning theory encompasses four elements arguing that engagement with social issues is fundamental to how learning takes place and how people become who they are (38). It should be noted that in practice the four elements are intertwined and reinforce each other. Though, in this section, the four elements are exemplified with a couple of examples from our experiences.

**Figure 9:**
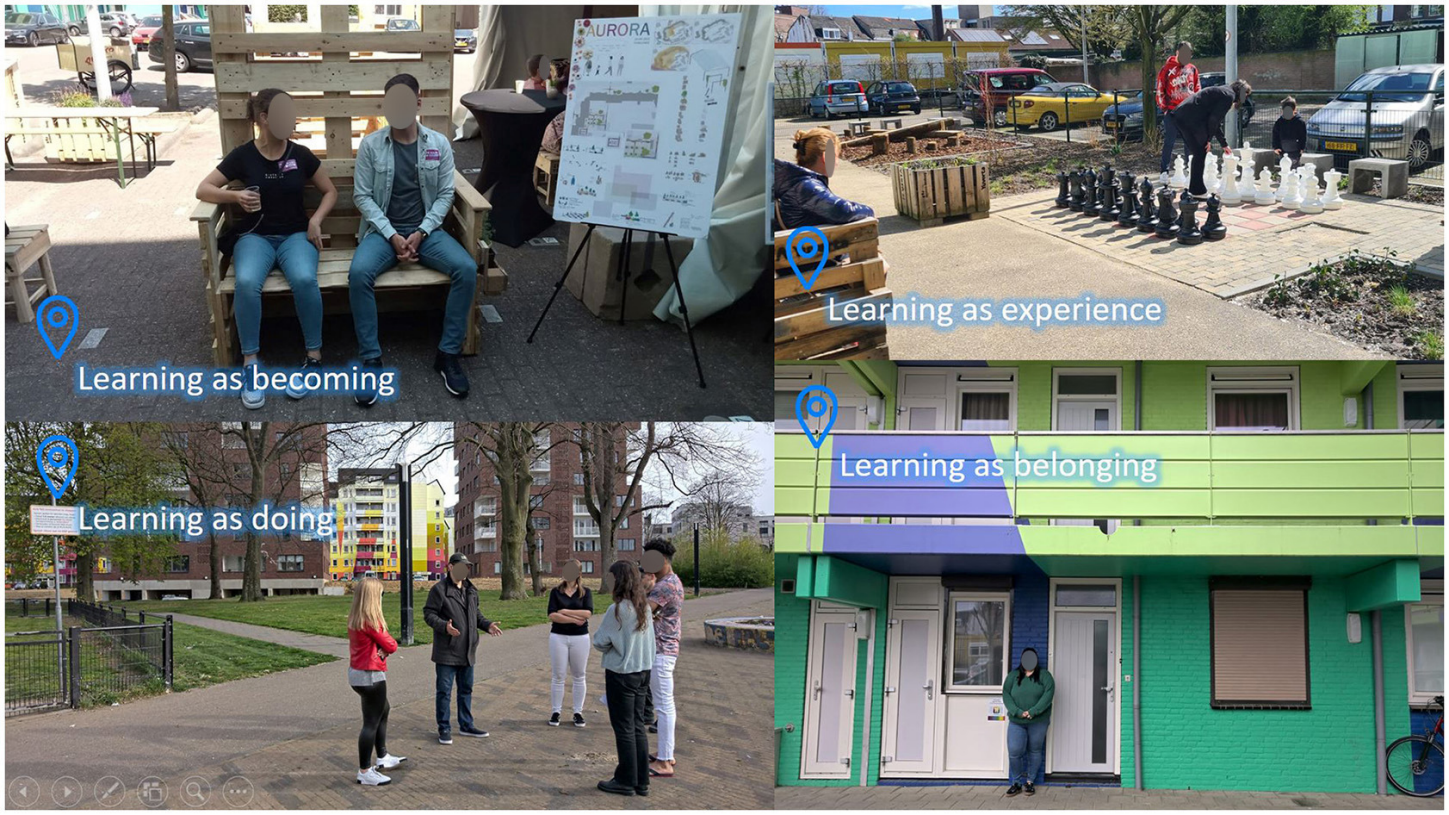
Social Learning in practice (Source: SURD).

1. *Learning as belonging*: students are part of the ULL community; they learn within and with the local stakeholders and community actors. As the Aurora apartment building process has been ongoing for almost 2 years now, it is observed that students, professional stakeholders, and citizens are taking ownership of the ULL, the Aurora courtyard, and local challenges. For example, third-year students who engaged themselves in the Aurora Challenge are actively asking partners like the housing association for internship positions or the SURD for graduation research positions in the current 4th year of their study. Similarly, citizens who co-hosted our photo group discussions during the Aurora Days are now actively supporting teachers in student assignment presentations to elaborate upon the historical context of the area. Finally, and from a community-perspective, the intern who lives in the Aurora apartment building is an actual part of the community and therefore a continuous presence both from the University of Applied Science—formal and personal—informal perspective.
2. *Learning as becoming:* Students collaborate with the ULL community to develop their own work identity relative to other disciplines. Here, a specific example of a spatial planning student is mentioned worthy to explain. The student participated in the Aurora Challenge while being on internship at a private project developer. While working on the Aurora Challenge and currently doing her graduation research at SURD, she developed herself as an architectural activist against hostile architectural practices by the municipality that forbid certain activities for citizens in public spaces. Her experiences in the ULL community made her aware of what she stands for and wants to stand for as a professional in the built environment discipline relative to other disciplines like health or nature; not as a private developer focusing on financial profit, but as a local activist ambitioning community wellbeing for citizens in their own living environment.
3. *Learning as experience*: Students learn by working on real-life societal issues in a local context. The context, and its undergoing changes, makes learning and working meaningful for students. In essence, the actual change achieved exemplifies this element. Students reported that they experience the ULL community and its focus and activities as extremely motivating and valuable due to the fact that they see the concrete change and improvement from their efforts for the local community and their wellbeing. In contrast to traditional classroom teaching, the student learning curve is enhanced by working across various disciplines, transversal educational institutes as well as actors covering the quadruple helix model and multiple development phases. The process also allows students to change roles with other students. When students are engaged long enough in the process, they gain experience that allows them to be coach and guide other newly involved students in group assignments. As such, the interplay between student and coach roles is an important professionalization experience. Similar impacts are observed among other stakeholders.
4. *Learning as doing:* Students learn to take initiative and responsibility regarding the societal issues and the ULL community to gain knowledge and skills and reflect on their own as well as other disciplines. Here, we refer to the intern duo who were responsible for the photo group discussions, posters, and Walk and Talk sessions and another occupational therapy student intern. First, the duo explained that they learned tremendously by organizing and hosting the mentioned activities in the “learning together” phase. By actually doing and working with the ULL community, planning the activities and necessities, they improved their skills and reflected upon the role of educational institutes in local urban transformation processes. Second, the occupational therapy student elaborated to one of the involved spatial planning teachers that the circular furniture was made from waste streams yet was not user-friendly from an occupational therapy perspective due to being limited in design by waste stream dimensions. This stimulated both the discussion on the role of occupational therapists in the built environment improvement and the discussion on the ergonomic aspects of the circular economy transition between student and researcher-teacher (Figure 9).

## 9 Conclusion: ULLs as infrastructure for urban innovation

To begin with, we want to highlight the novelty of our study in two ways.

First, the future is global. Global challenges and transitions are manifested in many different ways on a local level. To master these challenges and transitions, it is acknowledged that technological innovation alone is not sufficient, but rather requires social and regulatory innovation and infrastructures as well [see, e.g., (28)]. While we move toward the knowledge society and try to address these challenges and transitions, it is observed that local and transdisciplinary knowledge generation then is not motivated by cognitive knowledge or theories but rather by real-world needs for change and reform, like the Aurora courtyard and the health of its residents. In fact, this transdisciplinary learning comes both from the conceptual development and co-design of an urban intervention on study, i.e., an experiment, as well as the implementation, realization, and observation of a transformative experiment as aspired by the local community in practice. As West et al. (39) recently put it: “*the modus operandi of knowledge societies can then be understood as continuous experimentation”* (p. 136). Consequently, the ULL has proven to function as a social learning infrastructure that connects societal issues, research questions and methods, and education across various levels to enhance transdisciplinary learning among students, and also, local stakeholders about societal challenges. Subsequently, creating meaningful experiences and personal and professional growth among involved persons and parties, like residents, professionals and students. Therefore, the ULL is capable of operationalising and fulfilling the social learning theory potentials, i.e., transdisciplinary learning and action-oriented capacities and competencies like critical thinking (40), which seems so necessary and promising for today's sustainability transitions and challenges on local and global scales.

Second, the ULL functions as an alternative spatial planning and urban innovation approach to governing local neighborhood development in and for collective learning about a context of extreme urban and social conditions. ULLs do so due to their potential and ability to bridge a multitude of perspectives and disciplines as well as go beyond particular and traditional development phases only by the creation of a flexible process that is open for continuous evaluation, alteration, and improvement. It has proven to function as a kind of platform that is able to respond to short-term urgent needs, while at the same time, providing design scenarios and imaginative references for long-term development prospects. As such, it functions as an instrument to outline and accelerate placemaking processes and urban interventions that go beyond the design phases of urban development and explicitly experiment in practice by drawing upon local urban complexities for value creation, i.e. community wellbeing as aspired by the local community. The co-creation process and the urban intervention have positively impacted the community wellbeing. Residents, students, professionals, researchers, and others have been actively engaged in the transformation process leading to a stronger sense of community, sense of place, and local identity. Currently, we observe and notice increased levels of trust between stakeholders and ownership toward local issues and challenges. Thus, we argue that the explicit combination of placemaking and ULLs puts the potentials of placemaking, merely grown as a conceptual approach, into practice and *beyond* project-based operations. Hence, driving the pursuit of public health equity and community wellbeing impacts in neighborhoods.

## 10 Discussion

We elaborate upon two main points for our discussion.

First, we argued that placemaking, as a conceptual shift in urban planning, not only reshapes the physical environment but also reimagines the social-health fabric of these neighborhoods. We have seen, and by drawing on research in the public health domain, that by the creation of social interactions and community engagement, it is possible to address health disparities rooted in the (re)-production of the built environment. Our study in fact supports this claim, and indeed existing research provides indicators to assess the success of placemaking and the quality of public space [see, e.g., (41)]. However, while the process was designed by co-creation theory from Puerari et al. (17), we notice that literature in itself from Placemaking, ULLs and the Healthy City often refers to outcome indicators to focus on rather than process indicators that are important to create those wished-for outcomes. Therefore, we question whether the impacts of placemaking practices and ULLs from a public health domain perspective can rather be assessed and evaluated by outcomes or process indicators, especially when drawing upon the local community and unique local values as a main driver of the activities and knowledge generation, instead of political or policy motivations.

Second, while social learning theory guided the learning activities, one should note that the four elements remain rather vague and seen from an individual perspective. That is not to say it is ineffective as our results show the opposite, yet, the question arises: What challenges and enabling factors hinder or stimulate transdisciplinary learning in ULLs and both from an individual learning perspective as well as cross-sectoral collaboration constellation perspective? Consequently, it prompts the question of what role education and learning, and knowledge institutes like SURD, play in the knowledge society as well as its responsibility toward the transferability of practices and learning toward other contexts. Scholl, de Kraker and Dijk (42) hereto propose a meta-level approach to ULL learning and highlighting the de-contextualization and re-contextualization of learned lessons. However, in practice and from social learning theory and our experiences so far, it remains unclear on an operational level how to organize and facilitate this meta-learning.

Furthermore, we emphasize in an enumerative manner the implications for theory, policy, practice, and further research.

### 10.1 Implication for theory

Our experiences show three implications for theory:

1. The importance of local sustainability transitions and localized solutions. While global sustainability challenges negotiations and frameworks, like the SDGs, are crucial in overcoming them [see, e.g., (27, 29)], our experiences suggest that real-world transitions happen indeed on a local scale. Theories should therefore be tailored to address context-specific challenges and needs, rather than only emphasizing the importance of local contexts. As a consequence, it is needed to verify and enhance theories to bridge the gap between practice and existing theories and models on ULLs that are abstract and cannot be directly implemented in local contexts. As an example, the Harmonization Cube methodology (43) or the three-layer model (44) should be understood, tested, and adjusted more intensively in practice to help stakeholders on a local scale.
2. The integration of social learning theory elements in community wellbeing affairs and ULLs by bridging the gap in monitoring the impacts of ULLs beyond the context and extending it to diverse stakeholders engaged in the processes of ULLs. Currently, we notice that transdisciplinary knowledge production and learning are presented and viewed as an additional layer to ULLs rather than an integral part of it. Hence, we argue that social learning should be an integral part of addressing societal issues and challenges among all stakeholders involved. Thus, it is not only the students who learn, but all stakeholders that must be willing to learn in order to enhance impact creation. In specific, we argue to integrate social learning (38) in education and urban planning to emphasize the need for transdisciplinary learning and collaboration to effectively address societal challenges. Examples include the urban experiment, research-based education, or challenge-based learning (4).
3. The reconceptualisation of Urban Planning in practice by combining placemaking and ULLs. It is the exact integration of ULLs as an alternative urban planning methodology that is not strongly (if at all) presented in spatial planning theory. Planning theory contains this dichotomy of theories on the one hand and practice on the other, arguably top-down vs. bottom-up and/or technical vs. communicative planning theory (45). While placemaking conceptually grew as an alternative urban planning approach [see (12)], it is the combination with ULLs that puts its potentials into practice beyond project-based operations. The prioritization of, for example, community needs and wellbeing, social cohesion, or sense of place next to economic profits and planning philosophies can put urban planning at the center of improved quality of life as exemplified by Horstman and Knibbe (9) via advancing “place understanding” through a process of collaboration and interactive transdisciplinary learning among urban stakeholders.

### 10.2 Implication for policy

Our experiences show two implications for policy:

1. The need for political support for placemaking and ULLs. Policymakers should consider supporting, facilitating, and incentivising placemaking initiatives and ULL infrastructures on a local scale. Both offer the potential to bridge the gap between the quality of the built environment and community wellbeing, or the gap between exchange and user value (12), to align local actions with agreed global goals, like the SDGs. Indeed, Savini and Bertolini (46) show that this is crucial for the development pathway of ULLs in order to enhance impacts and Blezer and Abujidi (30) hinted toward similar support in that grant providers are challenged to rethink and redefine selection criteria for subsidy approvals on various political layers to enhance the transformative change capacity of ULLs.
2. The need for education reforms. Policymakers should consider reforms in higher education policies to align higher education with societal needs and sustainability goals. Social learning theory presents four elements that are important therefore, however are not sufficient by themselves. Other related concepts, like experiential learning theory, transdisciplinary learning, challenge-based learning, or real-world problem solving (4) are other perspectives. In fact, a recent publication [(47), p. 6] argues that “*a university living lab governance framework is needed to generate a culture of collaboration across research, teaching, operations, and enterprise and accelerate impact, without stifling emergence and innovation.”* While the authors provide recommendations like flexible coordination or relationship building between silos within the university that we acknowledge as well based on our experiences, we also add to this that there is a need to redefine the role of knowledge institutes in today's society, particularly from policymaker perspective. For example, the funding system of education (and research) in the Netherlands is currently based on quantitative measures. However, we do argue that qualitative measurements that are aligned with societal challenges and needs are required in order to guarantee quality education and research to address societal challenges and educate location-aware global citizens.

### 10.3 Implication for practice

Our experiences show four concrete implications for practice:

1. *Community engagement:* Practitioners in urban planning should prioritize community engagement and co-creation processes. Placemaking and ULLs demonstrate that involving local residents, students, professionals in the design, decision-making, and implementation of urban interventions can lead to more holistic and sustainable outcomes.
2. *Transdisciplinary collaboration:* Practitioners should foster transdisciplinary collaboration among students, researchers, professionals, and community members as this enables a more comprehensive place understanding of complex urban and societal challenges with responsive solutions.
3. *Experiential learning:* Educational institutions and practitioners should promote the inclusion of Social Learning dimensions. This includes students, teachers, and researchers to actively participate in addressing local experienced societal issues within their contexts and domains. As such, it helps to bridge the gap between scientific and theoretical knowledge with practical application.
4. *Tailored solutions:* Urban planning practitioners should tailor solutions to the specific needs and characteristics of the place that they will intervene in. Recognizing the uniqueness of places and their communities and involving them in the decision-making process can lead to more aspired and effective interventions.

### 10.4 Notes on further research

Our experiences call for further research along the following lines:

1. First, and process-wise, more experiences and insights are needed into the diverse roles that stakeholders can or must play based on the activity and phase in the ULL. In particular, when engaging multiple disciplines and education. For example, Vinke-de Kruijf et al. (48) provide a robust review of research roles in transdisciplinary projects. However, we observe and experience fluid roles during the process of urban interventions in ULLs that should be better understood to enhance effectiveness.
2. Second, and process-wise, we call for more intense, structured, and comprehensive monitoring and evaluation activities in ULLs. Our experiences made clear that impacts go beyond what individuals and stakeholders imagined and expected. We argue it is important to understand the full impacts of ULLs to better understand their innovation potentials. Consequently, we not only call ULL practitioners to focus on development activities but also more on impact monitoring and evaluation activities; both formally and informally as well as on the short term and the long term. Examples may be learning aspects or the maturity level of ULLs for impact creation.
3. Third, and content-wise, we call for more research in the urban planning and health domains to their interconnectedness, especially toward institutional causalities of public health equity in various geographical areas. While our experiences show and emphasize the importance of ULLs to the quality of the built environment in relation to health impacts (and other domains) on the local scale, it focuses on addressing observed societal issues; not the causalities of the societal issues in the first place. So, we argue that understanding the institutional arrangement through which health as a discipline on the one hand and urban planning as a discipline on the other influence each other mutually. It is crucial to understand these legal institutional causalities if ULLs as innovation infrastructures indeed want to scale up impacts for community wellbeing beyond the local scale and beyond the solution-focussed perspective.

## Data availability statement

The raw data supporting the conclusions of this article will be made available by the authors, without undue reservation.

## Ethics statement

Written informed consent was obtained from the individual(s) for the publication of any potentially identifiable images or data included in this article.

## Author contributions

SB contributed to the theoretical and practical conception and design of the study, wrote the first draft of the manuscript, contributed to manuscript revision, read, and approved the submitted version. NA and HS contributed to the theoretical and practical conception and design of the study, contributed to manuscript revision, read, additions, and approved the submitted version. All authors contributed to the article and approved the submitted version.
